# Early growth response protein 1 regulates promoter activity of *α*-plasma membrane calcium ATPase 2, a major calcium pump in the brain and auditory system

**DOI:** 10.1186/s12867-017-0092-1

**Published:** 2017-05-22

**Authors:** Rebecca R. Minich, Jin Li, Bruce L. Tempel

**Affiliations:** 1 Department of Pharmacology, School of Medicine, University of Washington, Seattle, WA 98195 USA; 2 Department of Otolaryngology-HNS, School of Medicine, University of Washington, Box 357923, Seattle, WA 98195 USA; 3 Virginia Merrill Bloedel Hearing Research Center, School of Medicine, University of Washington, Seattle, WA 98195 USA

**Keywords:** Atonal bHLH transcription factor 1 (ATOH1), Plasma membrane calcium ATPase 2 (ATP2B2), Plasma membrane calcium-transporting ATPase 4 (ATP2B4), Early growth response protein 1 (EGR1), Gene regulation, Gene transcription, Minimal promoter, Promoter

## Abstract

**Background:**

Along with sodium/calcium (Ca^2+^) exchangers, plasma membrane Ca^2+^ ATPases (ATP2Bs) are main regulators of intracellular Ca^2+^ levels. There are four ATP2B paralogs encoded by four different genes. *Atp2b2* encodes the protein pump with the fastest activation, ATP2B2. In mice, the *Atp2b2* transcript has several alternate transcriptional start site variants: *α*, *β*, *µ* and *δ*. These variants are expressed in developmental and tissue specific manners. The *α* and *β Atp2b2* transcripts are equally expressed in the brain. *αAtp2b2* is the only transcript found in the outer hair cells of young mice (Silverstein RS, Tempel BL. in Neuroscience 141:245–257, 2006). Mutations in the coding region of the mouse *Atp2b2* gene indicate a narrow window for tolerated dysfunction of the ATP2B2 protein, specifically in the auditory system. This highlights the necessity of tight regulation of this gene for normal cell physiology.

**Results:**

Although ATP2Bs are important regulators of Ca^2+^ in many cell types, little is known about their transcriptional regulation. This study identifies the proximal promoter of the *αAtp2b2* transcript. Further investigations indicate that ATOH1 and EGR1 modulate promoter activity. Additionally, we report that EGR1 increases endogenous expression of *Atp2b2* transcript in two cell lines. Electrophoretic mobility shift assays (EMSA) indicate that EGR1 binds to a specific site in the CpG island of the *αAtp2b2* promoter.

**Conclusion:**

This study furthers our understanding of *Atp2b2* regulation by: (I) elucidating transcriptional regulatory mechanisms for *Atp2b2,* and (II) identifying transcription factors that modulate expression of *Atp2b2* in the brain and peripheral auditory system and (III) allows for future studies modulating gene expression of *Atp2b2.*

**Electronic supplementary material:**

The online version of this article (doi:10.1186/s12867-017-0092-1) contains supplementary material, which is available to authorized users.

## Background

### Importance of Ca^2+^ and Ca^2+^ regulators

Ca^2+^ is involved in cellular excitation and is an important second messenger. In a resting cell, typical intracellular Ca^2+^ concentrations are between 0.1 and 1.0 μM, while extracellular levels are much higher (~2 mM). Consequently, maintaining this large electrochemical gradient is critical for normal cell physiology and necessitates an energy dependent mechanism of Ca^2+^ expulsion [1]. Plasma membrane Ca^2+^ ATPases are ubiquitously expressed in the plasma membrane and use ATP to pump Ca^2+^ out of the cell. Four paralogs are found in mammalian cells: *Atp2b1*, *Atp2b2*, *Atp2b3* and *Atp2b4*. These proteins are highly similar but differ in tissue expression and speed of activation.

### Animal and disease models associated with ATP2Bs

The importance of ATP2Bs for normal cellular function is demonstrated by widespread tissue expression, a pivotal role in disease, and the lethality of ATP2B null mice [1]. In mammals, ATP2B1 and ATP2B4 are the housekeeping Ca^2+^ regulators expressed throughout all tissues [2]. Null mutations of ATP2B1 are embryonic lethal and null mutations of ATP2B4 cause problems with sperm motility [3]. Genome wide association studies in humans have linked SNPs in ATP2B1 to increased cardiovascular risk [4] and a mutation in ATP2B4 was associated with spastic paraplegia [5]. ATP2B2 is expressed predominately in the brain and mammary glands. In mice, *Atp2b2* null mutations cause profound deafness and ataxia [6, 7]. Similarly, mutations in human ATP2B2 are associated with hearing loss [8, 9]. *Atp2b2* is also an emerging player in autism and breast cancer [10, 11].

### *Atp2b2* haploinsufficiency

ATP2B2 is highly expressed in the auditory system which is extremely sensitive to small physiological changes. This makes it an ideal model for studying fine tuning of Ca^2+^ regulation. The *deafwaddler* (*dfw*) mutant mice, *dfw* ^*2J*^ and *dfw* ^*i5*^, have null mutations in *Atp2b2* resulting in negligible expression of *Atp2b2* transcript and protein [6, 12]. Heterozygous mutants express about 50% normal protein levels and have significantly impaired hearing sensitivity compared to wild-type animals [12]. This finding suggests that one normal copy of *Atp2b2* is insufficient to produce a normal hearing phenotype and defines *Atp2b2* as haploinsufficient.

The original *dfw* mutation is a hypomorph that decreases, but does not completely abolish ATP2B2 activity. Studies of pump kinetics estimate that total function of ATP2B2 in homozygous *dfw* mutants is ~30% of normal animals [13]. Although homozygous mutants are profoundly deaf, heterozygous *dfw* mice do NOT have any significant hearing loss [14]. This is surprising considering the predicted increase in total pump function for *dfw* heterozygotes over the *dfw* ^*2J*^ and *dfw* ^*i5*^ heterozygotes is quite small. Thus, the threshold for ATP2B2 pump dysfunction in the auditory system is narrow and exhibits the necessity for tight regulation of this gene and protein.

### The importance of studying endogenous regulation of ATP2Bs

Although a wealth of information exists for post-transcriptional mechanisms regulating ATP2Bs, there is very little knowledge of transcriptional initiation of these genes. The only ATP2B promoter studied to date was reported in a brief publication outlining the promoter elements of *Atp2b1* [15]. There have been no studies looking at the promoter of the *Atp2b2* gene. It is clear that understanding the intricacies of ATP2B gene transcription will be important for combating the pathophysiology of diseases associated with this family of Ca^2+^ regulators. With over 30 different splice variants and four different genes, the ATP2B family is vast. The current study aims to characterize the promoter elements of the neuronal and hair cell-specific *α* transcript of *Atp2b2*, and identify important transcription factors that bind to and modulate expression of this gene.

## Methods

### Tissue culture

OC-1 and OC-2 cells [16] were grown at 33 °C and 5% CO_2_ in Modified Eagle Media with Glutamax (Gibco: 41090-036) supplemented with 10% fetal bovine serum and 50 U/ml of γ-Interferon. Neuro-2a (N2A) cells were grown at 37 °C and 5% CO_2_ in Dulbecco’s Modified Eagle Medium (Gibco) supplemented with 10% fetal bovine serum and 1% streptomycin and penicillin. HeLa cells were grown at 37 °C and 5% CO_2_ in Dulbecco’s Modified Eagle Medium (Gibco) supplemented with 10% fetal bovine serum and 1% penicillin/streptomycin.

### Cloning promoter constructs

PCR was used to amplify the ~5.5 kbp proximal promoter in three pieces off of genomic DNA from the DBA/2J inbred mouse strain. Next, exons of the 5′ untranslated region (UTR) were amplified from complimentary DNA (cDNA) of *dfw* ^*2J*^ brainstem. A synthetic NcoI cut site was inserted in the 5′ UTR at the translational start site. These four pieces were cloned into TOPO^®^ vectors (ThermoFisher Scientific) and sequenced. The four pieces were digested out of the TOPO^®^ vectors with the following restriction enzymes: I—*Hind*III and *Avr*II, II—*Avr*II and *Bss*SI, III—*Bss*SI and *Hpy*99I, IV—*Hpy*99I and *Nco*I (Table 1). These pieces were ligated into the pGL3 Firefly Luciferase Vector (Promega Corporation: E1751).

**Table 1:** Restriction enzyme identity and location of cut site for promoter truncation constructs

Restriction sites		
I	*Nco*I (I)	+572
II	*Bss*SI	−287
III	*Eco*RV	−855
IV	*Eco*R1	−2133
V	*Nco*I (II)	−2209
VI	*Avr*II	−3997
VII	*Hind*III	−5399

### Cloning transcription factor constructs

Transcription factor cDNAs were subcloned from Addgene plasmids (Addgene: ATOH1: 33333, EGR1: 11729 and GATA3: 1332) or amplified from cochlear or brainstem tissue of CBA/CaJ mice (USF1 and POU4F3). All cDNAs were ligated into the pcDNA vector with an internal ribosome entry site (IRES) followed by EGFP. The EGR1 cDNA contains an N-terminal Flag^®^ tag. EGFP expression was noted in cells transiently transfected with the transcription factor constructs. Transcription factor cDNA was quantified in the OC-1 and N2A cell lines at baseline and after transfection with the plasmids. All of the transcription factors were expressed at similar levels after transfection (Additional file 1: Table S1).

### Luciferase assays

OC-1 and OC-2 cells were plated 24 h before all transfections at a density of 4 × 10^4^ cells per well in a 24 well plate. Cells were harvested 48 h after transfection with passive lysis buffer and assayed using the dual luciferase reporter assay system (Promega: E1910) with a manual luminometer (Promega). Three biological replicates were assayed in each experiment. Data is the average of at least three experiments.

For promoter element identification: the *αAtp2b2* promoter constructs in the pGL3 vector were transfected at 400 ng/well into OC-1 or OC-2 mammalian cells. The renilla luciferase standard vector (Promega) was included at 5 ng/well and a DNA:transfection reagent ratio of 1:4 was used (Fugene HD, Promega: E2311).

For luciferase and transcription factor co-transfection assays: The *αAtp2b2* promoter construct [+572/−2133] was transfected (200 ng/well) with each pcDNA transcription factor vector: *Atoh1*, *Egr1*, *Gata3*, *Pou4f3*, OR *Usf1* (200 ng/well). For *Egr1* only, the pcDNA *Egr1* transcription factor vector was transfected at 200 ng/well with three of the *αAtp2b2* promoter construct truncations [+572/−287], [+572/−855], OR [+572/−2133] (200 ng/well). For all co-transfection assays: the renilla luciferase standard vector was included at 2.5 ng/well and a DNA:transfection reagent ration of 1:4 was used.

### qPCR assays

OC-1 and N2A cells were plated 24 h before transfection in 12 well plates. OC-1 cells were plated at a density of 8 × 10^4^ cells per well and N2A cells were plated at a density of 1 × 10^5^ cells per well. Transcription factor constructs were transfected at 800 ng/well using a 1:3 ratio of DNA:transfection reagent. Cells were incubated with constructs for 48 h and then harvested in 400 µl of QiAzol™ lysis reagent. mRNA was extracted using RNeasy Plus Universal Mini Kit (Qiagen: 73404) and subsequently converted into cDNA using random hexamer (ThermoFisher Scientific: N8080127) and reverse transcriptase (Clontech SMARTscribe: 639537).

Baseline expression experiments utilized primers designed to recognize specific cDNAs (Table 2). IQ™ SYBR^®^ Green Supermix (Bio-Rad: 170-8886) reagent was used. Endogenous levels of *Atp2b2* and *Atp2b4* transcript were quantified using premade gene expression assays from Applied Biosystems. (*Atp2b2*: Mm00437640_m1 and *Atp2b4*: Mm01285597_m1). TaqMan reactions were run using the SSOAdvanced™ Universal Probes Supermix (Bio-Rad: 172-5281). Expression levels were normalized to housekeeping genes, hydroxymethylbilane synthase (*Hmbs*) and γ-Actin (*Actg1*) (Applied Biosystems: *Hmbs*: Mm01143545_m1 and *Actg1*: Mm01963702_s1). All experiments were done on a Bio-Rad Q5 iCycler^®^ or CFX96 Touch™ (Bio-Rad). For all experiments, three biological replicates were assayed, data is the average of at least three runs where replicates within a run had a relative standard deviation of <0.03 (replicates with standard deviation/cycle threshold >0.03 were excluded).

**Table 2:** Primers used to quantify baseline levels of transcription factor mRNA message in the OC-1 cell line

Primer name	Primer sequence
*Actg F*	5′-GAA GGA GAT CAC AGC CCT AGCA-3′
*Actg R*	5′-GAC AGT GAG GCC AGA ATG-3′
*Hmbs F*	5′-CAG GCC ACC ATC CAG GTC-3′
*Hmbs R*	5′-GAA TGT TCC GGG CAG TGA TT-3′
*Atoh1 F*	5′-GAG TGG GCT GAG GTA AAA GAG T-3′
*Atoh1 R*	5′-GGT CGG TGC TAT CCA GGA G-3′
*Egr1 F*	5′-CCT ATG AGC ACC TGA CCA CA-3′
*Egr1 R*	5′-AGC GGC CAG TAT AGG TGA TG-3′
*Gata3 F1*	5′-AAC CAC GTC CCG TCC TAC TA-3′
*Gata3 F2*	5′-GGC TAC GGT GCA GAG GTA TC-3′
*Gata3 R*	5′-GAT GGA CGT CTT GGA GAA GG-3′
*Pou4f3 F*	5′-ATG CGC CGA GTT TGT CTC-3′
*Pou4f3 R*	5′-GGC TTG AAC GGA TGA TTC TT-3′
*Usf1 F*	5′-CTG AAA CCG AAG AGG GAA CAG-3′
*Usf1 R*	5′-GTT GGG GTC AGG AAA AGT GG-3′
*Usf1 F2*	5′-CAG GGC TCA GAG GCA CTA CT-3′
*Usf1 R2*	5′-GGG AAT AAG GGT GGG TCC T-3′

### EMSA assays

HeLa cells were grown to confluency in two T-75 cm^2^ flasks and extracted using trypsin. Cells were thoroughly washed and nuclear extracts were isolated using cell extraction buffer (ThermoFisher Scientific: FNN0011) supplemented with protease inhibitor (Sigma–Aldrich). Protein concentration was determined using a bicinchoninic acid assay (ThermoFisher Scientific: 23227). Double stranded DNA probes were made by boiling complimentary single stranded probes at a concentration of 25 µm in annealing buffer for 5 min in 1 l of boiling water. The boiling water was removed from the heat and the tubes were allowed to slow cool to room temperature. The canonical EGR1 positive control was designed based on the canonical consensus site for EGR1 (GCGGGGGCG) [17, 18]. The Santa Cruz EGR1 control probe was purchased from Santa Cruz Biotechnology (SC-2529). All other probes were developed using binding site predictions and consensus information from MotifMap, MatInspector and TFBIND. Biotin labeled probes were purchased from Integrated DNA Technologies (Table 3). EMSAs were performed using the LightShift™ Chemiluminescent EMSA protocol (ThermoFisher Scientific: 21048) with 18 µg of HeLa nuclear extract per 20 µl reaction. The reactions were incubated at room temperature for 30 min without the addition of the biotin labeled probe. Upon addition of the biotin labeled probes the reactions were incubated for another 30 min. *For the supershift assays*: 2 µg of antibody were added to the reaction and incubated for 30 min prior to the addition of biotin labeled probes. The EGR1 antibody was purchased from Abcam^®^ (Ab174509, Rabbit polyclonal to N-terminus) and the negative control antibody to ATP2B2 was purchased from ThermoFisher Scientific (PA1-915, Rabbit polyclonal). The reactions were electrophoresed in 0.5% TBE on 5% TBE polyacrylamide gels. The gels were run at 4 °C for 2.5–3 h at 60 V. Gels were transferred at 350 mA for 1 h at 4 °C to Biodyne™ B Nylon Membrane (Pierce: 77016), cross-linked for 15 min with 312 nm bulbs. Gels were visualized using the Chemiluminescent Nucleic Acid Detection Module (ThermoFisher Scientific: 89880). Each experiment included the standard shift assay and the test assay. Test assays include: self-competition assay (n = 5), modified EGR1 probe assay (n = 2) and the supershift assay (n = 4). Quantification was performed on blots with exposures between 5 and 10 min where none of the bands were saturated. For each blot, background intensity was subtracted from shift bands. In the self-competition assays, competition probe concentrations ranged from 16× to 600× the standard probe. Self-competition experiments including more than one competition probe concentration were averaged and counted as a single experiment.

**Table 3:** Probe sequence for shift assays

EGR1 probes	Sequence
[−71 to −98]	GCC CGA GGG GAG CGG GGG AGG AGA GAG C
Modified [−71 to −98] (Mod)	GCC CGA *A* GG *T* AG C *A* G GG *T * AGG AGA GAG C
Santa Cruz positive control (SC)	CGA CGC TGC GTG GGC GGA GCG GGG GCG A
Canonical positive control (Can)	GGA TCC AGC GGG GGC GAGCGG GGG CGA

The SC positive control probe was purchased from Santa Cruz Biotechnology. The Can probe was designed utilizing the canonical consensus sequence found on Motifmap (34). Predicted binding sites for EGR1 are underlined in the probe sequence. Bases in the modified probe (Mod) that differ from the [−71 to −98] probe are italicized

## Results

### Genomic modifications at *Atp2b2* indicate transcriptional hot-spots in mouse cerebellum

**Fig. 1:**
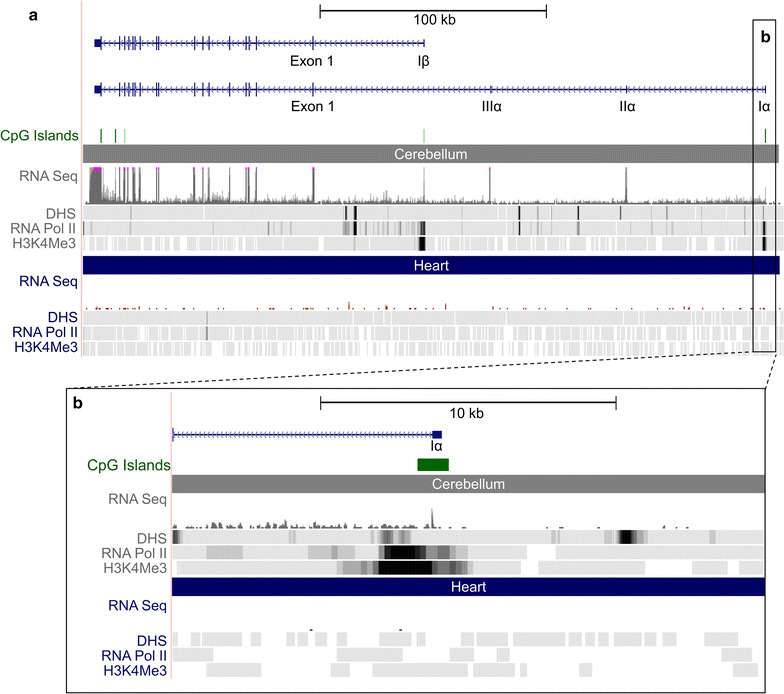
*Atp2b2* genomic region adapted from the UCSC genome browser. Image exported from UCSC genome browser (NCBI37/mm9), data is from the Encyclopedia of DNA elements (ENCODE) consortium [22, 23]. *Atp2b2* transcripts are shown in *blue*. **a** The whole genomic region surrounding *Atp2b2* is shown. Marks for transcriptional modulation are shown in 8 week mouse cerebellum vs 8 week mouse heart tissue. The RNA-Sequencing (RNA-Seq) data suggests that both the *α* and *β* transcripts of *Atp2b2* are expressed in the cerebellum. Neither transcript appears to be expressed in the heart. Both the *α* and *β* transcripts of *Atp2b2* have a CpG island at their TSSs. Markers of active transcription (DHS, RNA Pol II and H3K4me3) are present in the cerebellum (shown as a heat map) but are not present in the heart. **b** The region surrounding the *αAtp2b2* TSS is enlarged to show detail [23]

The genomic region surrounding *Atp2b2* is vast and complex. With 22 exons and four different transcriptional start site (TSS) variants, there are many opportunities for gene regulation. Of the four distinct *Atp2b2* TSS variants, *α* and *β* are the variants expressed in neurons. *αAtp2b2* is the primary transcript in the hair cells of young mice (postnatal day 9) [19]. The *α* and *β* transcripts share the same coding exons but differ in the sequence of their 5′ untranslated regions. Utilizing the UCSC genome browser, we compared the genomic regions of *α* and *β Atp2b2* in two distinct tissues to identify transcriptionally active domains. Chromatin features of interest included DNase I hypersensitivity (DHS), an indication of uncoiled, accessible DNA; RNA polymerase II (RNA Pol II) occupancy, the enzyme that transcribes DNA into RNA; and trimethylation of histone H3 at lysine 4 (H3K4me3), a hallmark of transcriptionally active promoters. The heat maps for these marks in the region surrounding the 5′ end of *Atp2b2* reveal several distinct signals in 8 week mouse cerebellum (indicative of active transcription) not detected in 8 week old heart [20] (Fig. 1). In particular, the regions directly surrounding the noncoding I*α* and I*β* exons contain significant levels of H3K4me3 and RNA Pol II occupancy and coincide with predicted CpG islands, known initiation sites for ubiquitously expressed genes [21]. Together, these data suggest that *αAtp2b2* and *βAtp2b2* transcripts are both transcribed in the cerebellum and have unique start sites. This allows for differential regulation and tissue specific expression. We have elected to focus on the *αAtp2b2* transcript because of its unique expression profile in the brain and sensory epithelium.

### Identifying the promoter of the *αAtp2b2* transcript

**Fig. 2:**
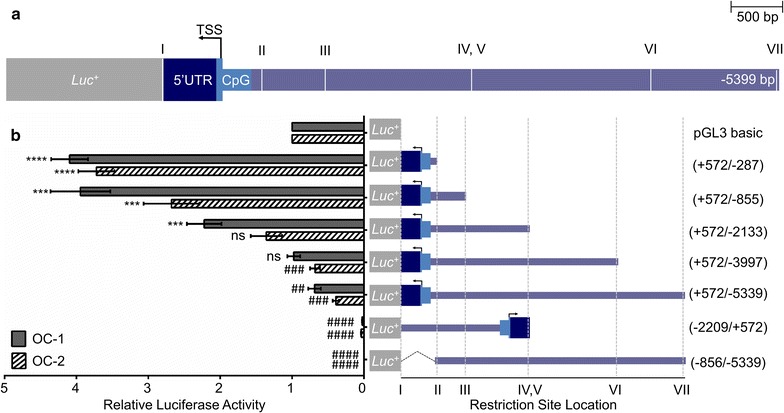
Promoter construct cartoon and luciferase assay. **a** *αAtp2b2* promoter elements and restriction sites. The gene for *Atp2b2* is located on the reverse strand oriented from *right* to *left*. This schematic represents the full promoter construct cloned into the luciferase vector. The full proximal promoter construct contains the three exons of the 5′ UTR (I*α*, II*α*, III*α*) and the 1st translated exon (Exon 1) up to the TSS (Table 4). Together these exons account for about 572 bases in the total construct. The rest of the 5.5 kbp construct contains the DNA directly upstream of the I*α* exon (*purple bar*). The CpG island is contained between +62 and −219 bases around the TSS (*blue bar*). The restriction sites used to create promoter construct truncations are indicated by *Roman numerals* on the cartoon, their exact locations can be found in Table 1. **b** The luciferase assay indicates that the minimal promoter elements are contained in the 5′ UTR and CpG island. The promoter is directional. Transcription is not initiated without the CPG island and the 5′ UTR. Activator elements are contained within the first 1.5–2.5 kbp of the promoter. Based on evidence from this figure elements upstream of 2.5 kbp seem to be inhibitory. Comparisons to the pGL3 empty luciferase vector were done using a Student’s *t* test. Data shown is the average of three biological replicates for at least three experiments, variation is shown as standard error of the mean (*asterisk* indicates mean greater than baseline, *hash* indicates mean less than baseline. *^,#^P ≤ 0.05, **^,##^P ≤ 0.01, ***^,###^P ≤ 0.001, ****^,####^P ≤ 0.0001)

The location of the CpG island and pattern of histone methylation in the cerebellum suggest that promoter elements are likely located directly upstream of the *αAtp2b2* TSS. To test this empirically, the 5′ UTR and 5 kbp region containing the CpG island were cloned into a luciferase reporter vector (Fig. 2a). All constructs are numbered relative to the TSS, which maps to the negative strand of chromosome 6 at base 114,042,026 (Ensembl version 82, GRCm38.p4). The “full-length” *αAtp2b2* promoter construct contains 5399 bases located directly upstream of the TSS from 114,042,027 [closest to TSS] to 114,047,365 [farthest from TSS]. Note that only the exons of the 5′ UTR were included in the construct, the 5′ UTR spans approximately 200 kbp of the genome (Table 4).

**Table 4:** Genomic location of the elements found in the full length proximal promoter construct

Construct elements	Genomic location	Construct location	Size (bp)
5' UTR			
Exon 1	113,842,332–113,842,645	+572 to +260	313
III*α*	113,920,509–113,920,603	+259 to +165	95
II*α*	113,980,542–113,980,587	+164 to +119	46
I*α*	114,041,909–114,042,026	+118 to +1	118
Proximal promoter	114,042,027–114,047,365	−1 to −5399	5399

Ten predominately neuronal or sensory-epithelium derived cell lines were screened for expression of *Atp2b2* transcripts (OC-K3, HEI-OCI, E-36, N2A, NIH/3T3, C2C12, OC-2, OC-1, ARPE-19, HeLa). *Atp2b2* gene expression was measured via quantitative PCR (qPCR) with a cutoff at 40 cycles. Under these conditions only two cell lines expressed detectable levels of *Atp2b2*, OC-1 cells from the immortomouse organ of Corti and the N2A neuroblastoma cell line. Given the origins of the other cell lines it is surprising that *Atp2b2* is not expressed; we speculate that this is an artifact of immortalization.

For the luciferase assay, we chose to use OC-1 cells and related OC-2 cells. These cell lines were immortalized during hair cell differentiation and, although OC-2 cells do not consistently express *Atp2b2,* OC-2 cells express hair cell markers that OC-1 lacks [24]. For this reason it was included in our luciferase assays. A series of *αAtp2b2* promoter luciferase constructs were transiently transfected into OC-1 and OC-2 cells and the fold change in promoter activity over empty pGL3 vector was determined (Fig. 2b). The greatest amount of luciferase activity above background was observed with the shortest promoter construct [+572/−287]. The inclusion of an additional 500 bp of 5′ sequence [+572/−855] had no detectable effect on promoter activity. When the length of the promoter fragment was extended further in the 5′ direction, the amount of luciferase activity began to decrease until it reached baseline levels. Reversing the orientation of the promoter or removal of the 5′ UTR and CpG islands completely abolished any detectable activity. These data demonstrate that the 5′ UTR and CpG islands are necessary and sufficient for promoter activity, and maps the minimal promoter between bases +572 and −287. The remaining proximal promoter region most likely contains important tissue specific enhancer and repressor elements.

### ATOH1, EGR1, GATA3, POU4F3, and USF1 are potential transcriptional modulators of *αAtp2b2*

**Fig. 3:**
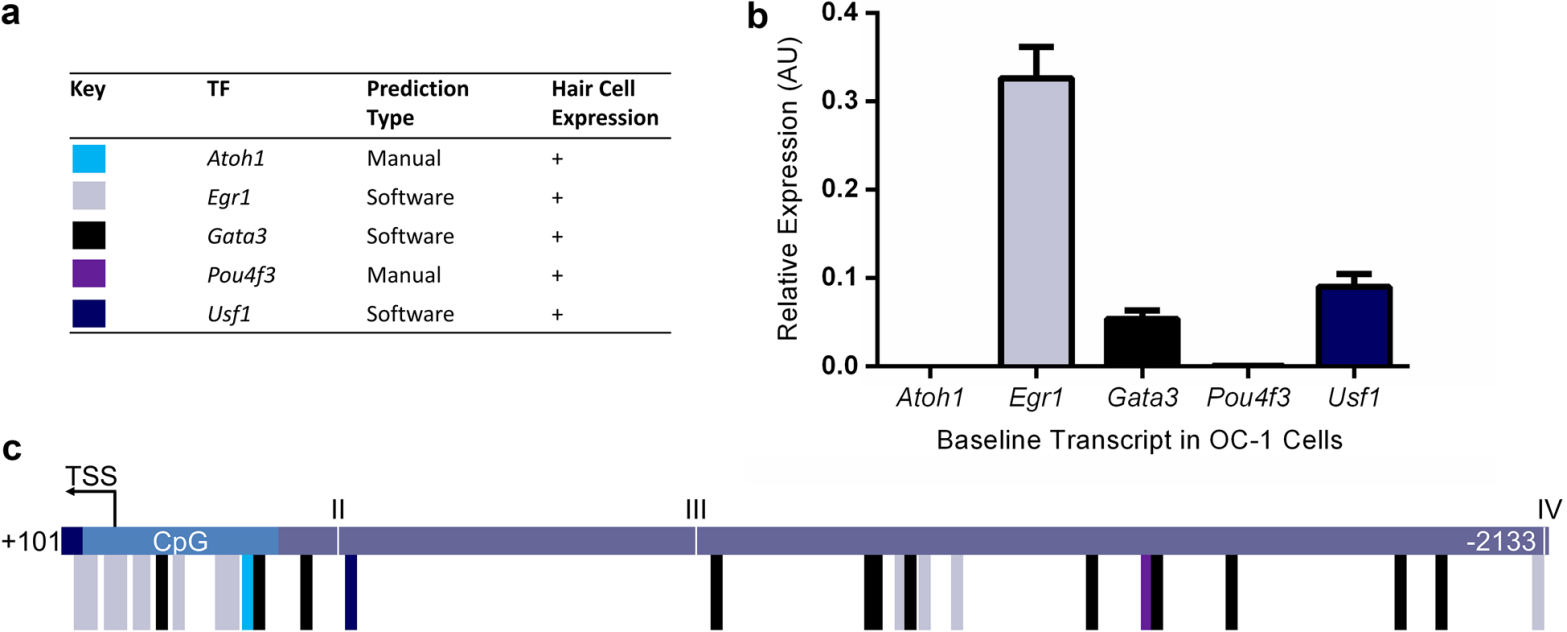
Transcription factors of interest and their binding sites in the *αAtp2b2* [+101/−2133] promoter. Transcription factors were selected based on a multi-faceted in silico search. **a** Predicted transcription factor binding sites were found using software (TFBIND and MatInspector) or manually identified using published consensus sequences [17]. Hair cell expression was determined utilizing SHIELD data [27, 39]. SHIELD RNA-Seq data was collected from FACS sorted GFP expressing hair cells, transcripts with reads above zero were considered expressed and denoted in the table with **a** (+). **b** Transcription factor expression in OC-1 cells was determined utilizing qPCR and cross-referenced with published microarray data. EGR1, GATA3 and USF1 are expressed [25–27, 40]. Data is the average of three technical replicates for three biological replicates, variation is shown as standard error of the mean. **c** Predicted binding sites for ATOH1, EGR1, GATA3, POU4F3 and USF1 are shown. Note the clustering of predicted binding sites for transcription factors in the CpG island

Binding prediction software, MatInspector and TFBIND [25, 26] were used to identify potential transcription factor binding sites in the proximal promoter of the *αAtp2b2* transcript. The entire region (+572 to −5399 bps) was analyzed and contained putative sites for thousands of transcription factors. To narrow down likely candidates we compared factors from the *αAtp2b2* promoter to the *βAtp2b2* proximal promoter and identified factors that were enriched in or unique to the *αAtp2b2* promoter. Using the Shared Harvard Inner Ear Laboratory Database (SHIELD) we determined expression of candidate genes in auditory hair cells [27] (Fig. 3a). We cross referenced these lists with transcription factor expression data for the OC-1 and OC-2 cells used in the luciferase assays. Published data for transcript expression in OC-1 cells was verified for select genes via qPCR (Fig. 3b). Two important hair cell factors, ATOH1 and POU4F3, were included based on manually identified binding sites in the proximal promoter (Fig. 3c). Candidates were narrowed down to the following transcription factors: ATOH1, EGR1, GATA3, POU4F3, and USF1. Together these transcription factors are involved in hair cell development and maintenance, synaptic plasticity, regeneration and tissue repair [28–38]. The predicted binding sites for these genes can be found in Fig. 3c.

### EGR1 and ATOH1 modulate the isolated *αAtp2b2* promoter in OC-1 cells

**Fig. 4:**
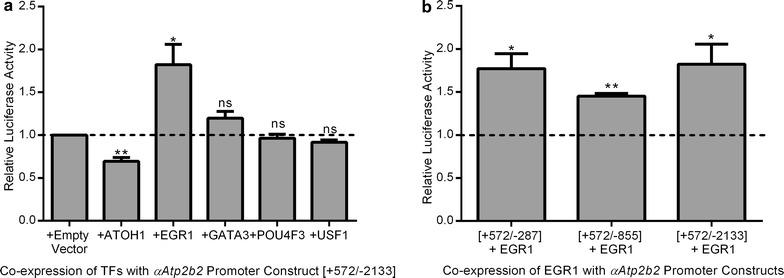
Co-expression of transcription factors with promoter constructs. **a** Luciferase activity of the *αAtp2b2* promoter construct [+572/−2133] when co-expressed with transcription factor constructs or empty vector. Luciferase activity of the [+572/−2133] *αAtp2b2* promoter construct co-expressed with transcription factor constructs was normalized to the luciferase activity of the [+572/−2133] promoter co-transfected with empty vector. Values were compared to a theoretical value of 1 utilizing a one-sample *t* test (*P ≤ 0.05, **P ≤ 0.01). **b** To narrow down the site of EGR1 activation in the *αAtp2b2* promoter, three promoter truncations were assayed. The promoter truncation constructs were co-transfected with EGR1 or empty vector. Luciferase activity of promoter constructs co-transfected with EGR1 were normalized to luciferase activity of the promoter construct co-expressed with empty vector. Normalized values were compared to a theoretical value of 1 using a one sample *t* test (*P ≤ 0.05 and **P ≤ 0.01). Activation occurs over baseline promoter activity in all three constructs suggesting that the EGR1 binding site is contained in the region of the CpG island. Data for the *αAtp2b2* promoter luciferase construct [+572/−2133] is the same in both **a** and **b**. Data shown is the average of three biological replicates for at least three experiments, variation is shown as standard error of the mean, *dashed line* represents the theoretical value of 1

To determine which of these transcription factors modulates the *αAtp2b2* transcript promoter, cDNA of the candidate transcription factors was cloned into a pcDNA vector upstream of IRES + EGFP. These vectors were co-transfected into OC-1 cells along with the [+572/−2133] *αAtp2b2* luciferase construct. Gene expression levels were similar for all of the transcription factors post-transfection (Additional file 1: Table S1). The [+572/−2133] construct was used because it is the longest promoter construct that has activity over baseline in the OC-1 cell line (Fig. 2b). Figure 3c shows a cartoon of the sequence from base +101 to base −2133 in detail with the location of transcription factor binding sites and the CpG island. This experiment reveals that ATOH1 decreases *αAtp2b2* promoter activity by 30% while EGR1 produces a significant twofold increase (Fig. 4a). Overexpression of GATA3, POU4F3 or USF1 had no significant effect on *αAtp2b2* promoter activity (Fig. 4a).

### Identifying the region of EGR1 activity in the *αAtp2b2* promoter

ATOH1 and EGR1 are capable of modulating the [+572/−2133] *αAtp2b2* luciferase construct in the OC-1 cell line, indicating functional significance in a mammalian cell model. The potential binding site of ATOH1 can be narrowed to a single site in the CpG island of the *αAtp2b2* promoter (Fig. 3c). Conversely, EGR1 has numerous predicted binding sites throughout the *αAtp2b2* promoter region. To map the location of functional EGR1 binding sites within the promoter, three different luciferase reporter constructs: [+572/−287], [572/−855], and [+572/−2133] were co-transfected with EGR1 or empty vector into OC-1 cells. All three constructs showed a significant increase in luciferase activity with co-expression of EGR1 (Fig. 4b). This data suggests that EGR1 binds the *αAtp2b2* promoter between bases +572 and −287. Due to limitations of restriction sites in this region, putative binding sites for EGR1 were not narrowed further. The putative binding sites for EGR1 are contained in the CpG island which is 80% GC. Due to limitations of PCR and sequencing under these conditions, mutagenesis was not performed [41].

### The effect of transcription factor overexpression on endogenous *Atp2b2* and *Atp2b4* transcript levels

The luciferase assay results indicate that EGR1 and ATOH1 have a functional effect on the isolated *αAtp2b2* transcript promoter. However, this observation does not indicate that ATOH1 and EGR1 effect endogenous levels of *Atp2b2* transcript expression. To test this, EGR1 and ATOH1 were overexpressed in N2A and OC-1 cells that transcribe *Atp2b2* endogenously. Gene expression levels were similar for all of the transcription factors after transfection (Additional file 1: Table S1). Due to the low basal levels of *Atp2b2* expression, TaqMan assays with high target specificity and accuracy were utilized for this experiment. The TaqMan probes do not discriminate between the different alternate start site variants of *Atp2b2* so total *Atp2b2* transcript levels were quantified. *Atp2b4* transcript expression was also measured because there is evidence that *Atp2b4* compensates for decreased expression of *Atp2b2* in the stereocilia of *Atp2b2* mutant mice [14].

**Fig. 5:**
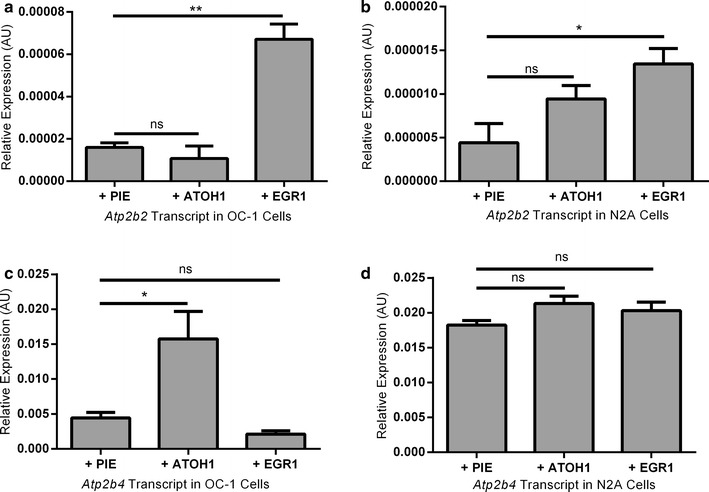
Effect of overexpression of Atoh1 and Egr1 on endogenous levels of *Atp2b2* and *Atp2b4*. **a**, **b** EGR1 increases expression of *Atp2b2* in both OC-1 and N2A cell lines. ATOH1 moderately inhibits expression of *Atp2b2* in OC-1 (ns) and has no effect in N2A cells. **c**, **d** *Atp2b4* transcript expression is unaffected by overexpression of EGR1 in both OC-1 and N2A cells. ATOH1 increases expression of *Atp2b4* transcript in OC-1 cells but not in N2A cells. Comparisons to empty vector (PIE) were made using a Student’s *t* test (*P ≤ 0.05, **P ≤ 0.01). Data shown is the average of three technical replicates for three biological replicates in each cell line, variation is shown as standard error of the mean

In OC-1 and N2A cells, EGR1 overexpression increases transcript levels of *Atp2b2* recapitulating our luciferase results (Fig. 5a, b). When ATOH1 is overexpressed in OC-1 and N2A cells, *Atp2b2* transcript levels are unaffected (Fig. 5a, b). Although ATOH1 overexpression did not significantly inhibit *Atp2b2* transcript, *Atp2b4* levels were found to increase 3.5 fold over baseline in OC-1 cells (Fig. 5c). An ATOH1 driven increase in *Atp2b4* transcript was not observed in N2A cells (Fig. 5d). Given that ATOH1 is specific to developing hair cells it is unsurprising that there is a cell-type specific effect. EGR1 overexpression has no significant effects on *Atp2b4* expression in OC-1 or N2A cells (Fig. 5c, d). The luciferase data taken together with *Atp2b2* and *Atp2b4* expression data suggest interplay between EGR1 and ATOH1 in OC-1 cells. EGR1 increases *Atp2b2* expression while ATOH1 inhibits the α*Atp2b2* promoter and increases *Atp2b4* expression (Figs. 4a, 5a, c).

### Shift and supershift assays indicate that EGR1 binds to the *αAtp2b2* promoter

**Fig. 6:**
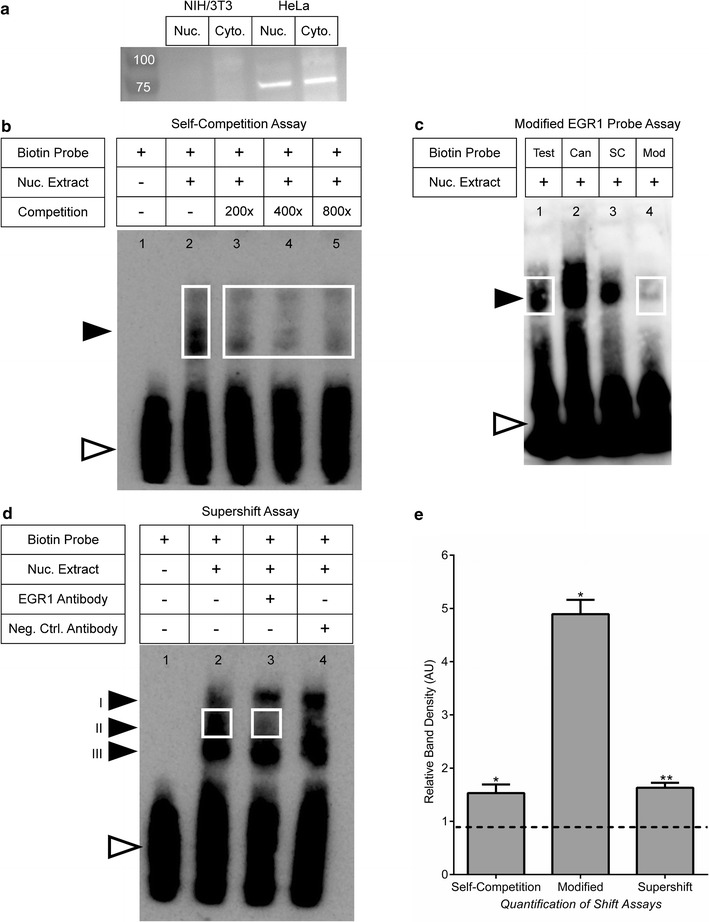
Shift assays of EGR1 binding to probe [−71 to −98] derived from the *αAtp2b2* transcript promoter. All blots shown are 10 min exposures. **a** Western blot of EGR1 protein expression in NIH/3T3 or HeLa cells. Nuclear and cytoplasmic fractions were compared. HeLa nuclear extract was used for shift assays due to its detectable expression of EGR1. **b** The [−71 to −98] probe was used in this assay labeled with biotin (Biotin Probe) or unlabeled as self-competition (Competition) *Lane 1*—free unbound biotin labeled probe is shown and is indicated by an *open arrow head*. *Lane 2*—contains HeLa nuclear lysate, a shift is indicated by *closed arrowhead*. *Lanes 3*–*5*—self-competition with unlabeled probe at increasing concentrations. **c** The [−71 to −98] probe (Test), two control probes (Can and SC) and the Modified [−71 to −98] (Mod) probes were used in this assay. *Lanes 1*–*3* positive shifts for the [−71 to −98] probe as well as two positive control probes designed to bind EGR1 (Table 3). *Lane 4*—[−71 to −98] modified probe, four nucleotides in the EGR1 cluster region were modified and the shift is abolished. **d** The [−71 to −98] probe was used in this assay (Biotin Probe). *Lane 1*—shows free unbound biotin labeled probe which is indicated by the *open arrow head*. *Lane 2*—contains HeLa nuclear lysate and recapitulates the shift from 6B/C. There are three binding populations in the shift indicated by *closed arrow heads* (I, II, III). *Lane 3*—reaction contains polyclonal IgG antibody targeting EGR1 along with HeLa nuclear lysate. Addition of the anti-EGR1 abolishes the binding of the second population to the probe (II). *Lane 4*—reaction contains HeLa nuclear lysate and polyclonal IgG antibody targeting ATP2B2. This antibody does not abolish the II shift and acts as a negative control for the supershift observed in *Lane 3*. **e** Average pixel density of the standard shift was normalized to the average pixel density of the test shift for each experiment (self-competition, modified EGR1 probe assay and supershift assay). *White boxes* indicate the shifts being compared in each experiment. Normalized values were compared to a theoretical value of 1 using a one sample *t* test (*P ≤ 0.05 and **P ≤ 0.01). All results are significant

It is likely that both EGR1 and ATOH1 play a role in modulation of *Atp2bs.* However, ATOH1 did not significantly affect endogenous levels *Atp2b2* in OC-1 and N2A cells. Given this negative data, only EGR1 was included in the binding assays. To identify potential EGR1 binding sites, data from MatInspector was cross-referenced with TFBIND software predictions. Sites with matrix similarity of 0.75 or higher (highest predicted binding being 1.0) were included. This identified 12 overlapping binding sites that could be divided into seven different regions of the promoter (Fig. 3). Of particular interest are three sites in the CpG island near the TSS between base −71 and base −98 (Fig. 3). A probe targeting this region was designed to experimentally test the protein binding potential of this region. Two positive control probes were used to verify the shift assay, one derived from the EGR1 consensus sequence (GCGGGGGCG) [17, 18] and a gel shift probe commercially available from Santa Cruz Biotechnology (Table 3). EGR1 protein levels were measured in OC-1, HeLa and NIH/3T3 cells by immunoblotting. Protein expression of EGR1 was not detectable in OC-1 cells (data not shown) or in NIH/3T3 cells (Fig. 6a). EGR1 was found in the nucleus and cytoplasm of HeLa cells (Fig. 6a).

All EMSAs were performed using HeLa cell nuclear extracts (Fig. 6b, c). No protein binding activity was detected with two test probes for sequences either downstream or at the TSS (data not shown). A mobility shift is only observed with the DNA probe derived from the site upstream of the TSS [−71 to −98] (Fig. 6b). Formation of this shifted DNA–protein complex is inhibited when increasing concentrations of unlabeled [−71 to −98] DNA probe are added as competitor. Four nucleotides in EGR1 binding cluster of the [−71 to −98] DNA probe were modified to investigate sequence specificity of binding (Table 3). This modified probe (Mod) is unable to produce a shift when compared to the [−71 to −98] probe (Test) derived from the *αAtp2b2* promoter and two positive control probes (Can and SC) (Fig. 6c). These experiments indicate that the shift is sequence specific but does not indicate which protein is binding to the probe.

To determine if EGR1 is the protein responsible for the mobility shift in Fig. 6a and b, antibody to EGR1 was added to the binding reactions. For these assays, the shifted complexes resolved into three distinct populations (Fig. 6d). The formation of complex II was dramatically inhibited upon addition of the EGR1 specific antibody but not the negative control antibody targeting ATP2B2 (Fig. 6d, *Lane 4*). These results suggest that EGR1 is binding to the [−71 to −98] probe in a sequence specific manner to stimulate *αAtp2b2* promoter activity. Although there are different populations (I and III) that remain bound to the probe in the presence of EGR1 antibody it is likely that this is non-specific binding or it is binding of the probe to a protein besides EGR1. There are a number of predicted SP1 binding sites in the CpG island. Previously published experiments have exhibited the similarities between binding sites for SP1 and EGR1 [18, 42]. It is plausible that this is the identity of the protein that remains bound to the probe in the supershift assay (Fig. 6d, *Lane 3*).

Densitometry was employed to compare the standard shift assay against the test shift assay for each of the above experiments: self-competition, modified EGR1 probe and supershift. The average pixel density of the standard shift was significantly darker than the test shift in all three experiments (Fig. 6e). This quantification supports the conclusion that EGR1 binds to the [−71 to −98] probe in a sequence specific manner.

## Discussion

### Initiation of gene transcription in *Atp2bs* and *αAtp2b2*

The Ca^2+^ ATPases play an important role in maintaining cell homeostasis but little is known about initiation of transcription of these genes. *Atp2b2* has the fastest activation and is one of the primary ATP pumps found in the brain and sensory epithelium. Investigations of this gene have highlighted its pivotal role in normal function of the auditory system as well as the vestibular system [19, 6, 7, 12, 43, 44]. Of the two transcripts found in the brain (*α* and *β*) only the *α* transcript is found in the auditory hair cells of young mice (postnatal day 9). This study focuses on the *α* transcript because of its pivotal role in the normal function of the auditory system.

### Identification of the *αAtp2b2* minimal promoter and repressor elements

Studies of the genomic region surrounding the *Atp2b2* gene indicate that the minimal promoter of the *α* transcript is likely located directly adjacent to the TSS. Our luciferase assay data narrows this to the region 2133 bp upstream of the TSS. The abolishment of promoter activity upon removal of the CpG island and the 5′ UTR narrows the minimal promoter to the 287 bps upstream of the *αAtp2b2* TSS. In both OC-1 and OC-2 cell lines, activity decreases as the promoter is extended in the 5′ direction. This is likely due to repressor elements upstream of the minimal promoter. Interestingly, the activity of the promoter falls off faster in OC-2 cells suggesting cell type specific repression and modulation of the promoter.

### The effect of ATOH1 and EGR1 on *Atp2b2*

In OC-1 cells, we find that ATOH1 decreases *αAtp2b2* proximal promoter activity by ~30%. However, we did not detect a significant effect of ATOH1 on endogenous levels of *Atp2b2* in N2A or OC-1 cells. This contradicts the luciferase assay which predicts that ATOH1 would inhibit endogenous levels of *Atp2b2* in OC-1 cells. A caveat of this result is that *Atp2b2* is very close to the limit of detection in OC-1 cells making any decrease in expression challenging to measure. It is therefore possible that ATOH1 does inhibit endogenous levels of *Atp2b2* in OC-1 cells but we were unable to detect it. This caveat does not apply to the EGR1 overexpression assay because EGR1 increases expression of *Atp2b2* in OC-1 cells (and in N2A cells). Any increase in *Atp2b2* expression is in the detectable range of the assay. In addition to increasing endogenous levels of *Atp2b2* in OC-1 and N2A, EGR1 also increases the activity of the *αAtp2b2* promoter twofold over empty vector. We conclude that EGR1 increases promoter activity of *αAtp2b2* and expression of *Atp2b2* transcript in OC-1 and N2A cells while ATOH1 represses promoter activity of *αAtp2b2* in OC-1 cells.

### The interplay of EGR1, ATOH1, *Atp2b2* and *Atp2b4*

In OC-1 cells, overexpression of ATOH1 increased *Atp2b4* transcript 3.5 times over baseline. However, the effect was not recapitulated in N2A cells. Our data suggests that in organ of Corti derived cells ATOH1 causes increased expression of *Atp2b4.* SHIELD data indicates that ATOH1 is expressed throughout development in hair cells with a peak around postnatal day 0 [27] (Table 5). Although SHIELD data shows only low *Atp2b4* expression, other studies have exhibited expression of *Atp2b4* in the hair cells at postnatal day 12 [45]. This expression may be driven by ATOH1.

**Table 5:** Developmental RNA-Seq data from the SHIELD database is shown

Gene	E16	P0	P4	P7
*Egr1*	18,501	73,459	19,502	13,120
*Atoh1*	386	1974	498	171
*Atp2b2*	3202	7861	11,109	16,536
*Atp2b4*	3	3	1	1

Normalized transcript reads are from FACS sorted mouse hair cells expressing GFP https://shield.hms.harvard.edu/datasets.html [27, 39]. Although *Atp2b4* expression is low, it is highest when *Atoh1* peaks at P0. *Atp2b2* expression starts to ramp up as *EGR1* peaks and after *Atoh1* goes down

Our data shows that EGR1 increases expression of *Atp2b2* while ATOH1 represses the *αAtp2b2* promoter. SHIELD data indicates that EGR1 levels are high in the hair cells of mice at embryonic day 16 and remain high with a peak at postnatal day 0. *Atp2b2* expression steadily rises through all of the ages assayed and it is possible that *Atp2b2* levels are inhibited in early development by ATOH1 and rises as ATOH1 turns off (Table 5).

This exhibits an interesting interplay between *Atp2b2* and *Atp2b4*. It is known that ATPases compensate for each other in knockout animals [3]. Notably, in *Atp2b2* null mice, *Atp2b4* is expressed abnormally in stereocilia into adulthood [44]. In OC-1 cells, ATOH1 increases expression of *Atp2b4* and represses the promoter of *Atp2b2*. This suggests that ATOH1 may be a developmental switch for Atp2bs in hair cells. For example, as ATOH1 turns off it may allow for *Atp2b2* to be turned on.

### The significance of EGR1 and *Atp2b2*

Although EGR1 increases expression of *Atp2b2* in both OC-1 and N2A cells, the low level of *Atp2b2* expression in both cell lines suggests there are other factors involved in transcriptional activation of this gene. This is unsurprising given the complexity of this gene and the necessity for tight control of *Atp2b2* for normal physiology. The effect of EGR1 seems specific to *Atp2b2* as overexpression did not increase expression of the closely related *Atp2b4* gene in either OC-1 or N2A cells. Investigation of the proximal promoter of *Atp2b4* on the UCSC genome browser suggests this is due to the lack of a CpG island. EGR1 is important for neuronal and sensory systems through involvement in synaptic plasticity, retinal formation and acoustic trauma. Additionally, it is implicated in auditory hair cell regeneration pathways [33–38].

## Conclusions

This is the first investigation of the *Atp2b2* promoter and is the second investigation of a promoter in the vast *Atp2b* family. This study identified the minimal promoter elements of *αAtp2b2* including a CpG island. Additionally, our investigations indicate that ATOH1 and EGR1 modulate *αAtp2b2* promoter activity. There appears to be interplay between EGR1, ATOH1, *Atp2b2* and *Atp2b4* in OC-1 cells; further studies are necessary to confirm these interactions and determine their physiological relevance in vivo. EGR1 was also found to bind to a specific sequence 71 bases upstream of the TSS in the CpG island of the *αAtp2b2* promoter. In conclusion, we have elucidated transcriptional regulatory mechanisms for *Atp2b2,* identified transcription factors that modulate expression of *Atp2b2* in the brain and peripheral auditory system, and established a foundation for future studies investigating expression of *αAtp2b2*.

## Additional file

**Additional file 1: Table S1.** Change in Transcription Factor Expression in OC-1 and N2A cells After Transfection. Normalized expression of transcription factors (AU) in OC-1 and N2A cells at baseline and after transfection with transcription factor constructs.

## Abbreviations

TSS: transcriptional start site
*Atp2b*: plasma membrane calcium ATPase
UTR: untranslated region
DHS: DNase I hypersensitivity
qPCR: quantitative PCR
RNA Pol II: RNA polymerase II
H3K4me3: histone H3 lysine 4 trimethylation
RNA-Seq: RNA-sequencing
EMSA: electrophoretic mobility shift assay
ATOH1: atonal bHLH transcription factor 1
EGR1: early growth response protein 1
POU4F3: POU domain class 4 transcription factor 3
GATA3: trans-acting T cell-specific transcription factor GATA3
USF1: upstream stimulatory factor 1
Ca^2+^: calcium
*dfw*: *deafwaddler*
cDNA: complimentary DNA
IRES: internal ribosome entry site
*Hmbs*: hydroxymethylbilane synthase
Gamma (γ) actin: *Actg1*
