# Supplementary material for: Early growth response protein 1 regulates promoter activity of α-plasma membrane calcium ATPase 2, a major calcium pump in the brain and auditory system
Source: BMC Mol Biol. 2017 May 22;18:14. doi: 10.1186/s12867-017-0092-1 (PMC5441030; doi:10.1186/s12867-017-0092-1)
Supplement: Supplementary file 1 — Additional file 1: Table S1. Change in Transcription Factor Expression in OC-1 and N2A cells After Transfection. Normalized expression of transcription factors (AU) in OC-1 and N2A cells at baseline and after transfection with transcription factor constructs. [file 12867_2017_92_MOESM1_ESM.docx]

| Gene | OC-1 Baseline | OC-1 + TF Construct | Fold Change |
| --- | --- | --- | --- |
| *Atoh1* | 1.95E-05 | 7.70 | 3.95E+05 |
| *Egr1* | 3.60E-02 | 3.58 | 9.96E+01 |
| *Gata3* | 6.22E-03 | 3.10 | 4.98E+02 |
| *Pou* | 4.11E-05 | 1.49 | 3.63E+04 |
| *Usf1* | 1.31E-02 | 1.11 | 8.48E+01 |
| **Gene** | **N2A baseline** | **N2A + TF construct** | **Fold change** |
| *Atoh1* | 5.12E-08 | 5.02 | 9.82E+07 |
| *Egr1* | 4.48E-03 | 2.81 | 6.26E+02 |

Supplemental Table 1 – Normalized expression of transcription factors (AU) in OC-1 and N2A cells at baseline and after transfection with transcription factor constructs. Fold change is included for reference. All genes were normalized to the expression of γ-Actin.
